# Clarifying Exercise Addiction: Differential Diagnosis, Co-occurring Disorders, and Phases of Addiction

**DOI:** 10.3390/ijerph8104069

**Published:** 2011-10-21

**Authors:** Marilyn Freimuth, Sandy Moniz, Shari R. Kim

**Affiliations:** School of Psychology, Fielding Graduate University, Santa Barbara, CA 93105, USA; E-Mails: smoniz180@gmail.com (S.M.); skim@email.fielding.edu (S.R.K.)

**Keywords:** exercise addiction, phases of addiction, behavioral addictions, co-addictions

## Abstract

This paper sets out to clarify the unique features of exercise addiction. It begins by examining how this addiction can be distinguished from compulsions and impulse control disorders both of which, like an addiction, involve excessive behavior that creates adverse effects. Assessment of exercise addiction also requires that clinicians be attuned to other forms of excessive behavior, especially eating disorders that can co-occur with exercise. Finally in an effort to clarify exercise addiction, this paper uses the four phases of addiction to examine the attributes of exercise that define it as a healthy habit distinct from an addiction. The paper ends with a discussion of the implications of these topics for effective assessment and treatment.

## 1. Introduction: Exercise as an Addiction

The upcoming Diagnostic and Statistical Manual of Mental Disorders (DSM-5) [1] will include behavioral addictions. Although gambling will be the only designated behavioral addiction, this new diagnostic nomenclature will no doubt lead to increased research into all forms of excessive behavior, such as exercise, that have been considered to be addictive. This research will require a clear description of exercise addiction as distinct from a healthy habit [2]. As with other behavioral addictions, it will also be necessary to distinguish exercise addiction from compulsions and impulse control disorders. An understanding of common co-occurring disorders will also be important to the extent that they mask exercise addiction and/or complicate treatment. The following presentation is based on accessing the CORK bibliography on exercise addiction and an extensive PsycINFO search of the topic.

### Defining Exercise Addiction

What distinguishes the everyday gym enthusiast from someone addicted to exercise? Would we consider an elite athlete training for the Olympics as having an exercise addiction? What about the devoted runner who adds an extra three miles to his or her running schedule after lunch at a fast food restaurant? Hausenblas and Downs [3,4] identify exercise addiction based on the following criteria that are modifications of the DSM-IV TR [5] criteria for substance dependence:

- *Tolerance*: increasing the amount of exercise in order to feel the desired effect, be it a” buzz” or sense of accomplishment;
- *Withdrawal*: in the absence of exercise the person experiences negative effects such as anxiety, irritability, restlessness, and sleep problems [6];
- *Lack of control*: unsuccessful at attempts to reduce exercise level or cease exercising for a certain period of time;
- *Intention effects*: unable to stick to one’s intended routine as evidenced by exceeding the amount of time devoted to exercise or consistently going beyond the intended amount;
- *Time:* a great deal of time is spent preparing for, engaging in, and recovering from exercise;
- *Reduction in other activities*: as a direct result of exercise social, occupational, and/or recreational activities occur less often or are stopped;
- *Continuance:* continuing to exercise despite knowing that this activity is creating or exacerbating physical, psychological, and/or interpersonal problems.

Although others have defined exercise addiction using different models [7–11], the above definition is most closely aligned with the DSM-5 criteria for behavioral addiction which will be modeled after those for substance dependence [1]. Based on a review of a wide range of studies on exercise addiction, Sussman, Lisha, and Griffiths [12] estimate the prevalence in the general population to be close to 3%. Among certain groups such as ultra-marathon runners [8] and sport science students [10] the figure is even higher. According to Lejoyeux, Avril, Richoux, Embouazza, and Navoli [13], 42% of the members at a Parisian fitness club met criteria for exercise addiction.

## 2. Results and Discussion

### 2.1. Distinguishing Exercise Addiction from Other Disorders

If research on exercise addiction is to move ahead, it will be important to know when this behavior actually represents an addiction and not some other disorder. Like other behavioral addictions, exercise addiction is often referred to as being compulsive or impulsive. This paper will address the overlaps among exercise addiction, compulsions, and impulse control disorders. Exercise addiction also needs to be distinguished from exercise that occurs at a high frequency. The Olympic athlete may devote a great deal of time to the activity, experience a significant reduction in other activities and go through withdrawal when the behavior is stopped or cut back. Despite meeting three exercise addiction criteria [3], an elite athlete is not necessarily addicted to his or her sport. Failure to distinguish exercise addiction from exercise done with high frequency and intensity has been a source of confusion in the literature [8,14]. Phases of addiction [2] will be used to distinguish exercise addiction from other forms of intense and frequent exercise behavior. Finally, exercise addiction co-occurs with other addictions that, if left unrecognized, can complicate the treatment process. The frequent link between exercise addiction and eating disorders will be emphasized.

### 2.2. Exercise Addiction, Exercise Compulsion, or Impulse Control Disorder?

Addictive behavior often is described as impulsive [15]. Impulsivity consists of rapid, unplanned responses to external or internal stimuli. Impulsive behavior is without sufficient contemplation for possible negative consequences and is primarily driven by a desired positive reward [16]. Exercise is a pleasurable activity that, in its addicted form, can occur without full consideration of negative consequences. For example, the addicted runner enjoys this activity and goes for a run despite knowledge of an impending rainstorm that increases the chance of injury. However, unlike an impulse-control disorder there is often considerable thought that precedes the action of engaging in an addiction. Like others who are addicted, the person addicted to exercise often considers the negative consequences but ultimately ignores them [17]. Furthermore addictive behaviors, unlike impulse-control disorders, develop tolerance and withdrawal.

Like other addictive behaviors, excessive exercise often is described as compulsive by theorists [18] and those highly involved with exercise [19]. But is this really a compulsive disorder or simply a term to describe the compelling nature of an addiction? A compulsive disorder consists of ritualized and stereotyped behaviors of which the most common examples are frequent checking and hand-washing [19]. Intrusive thoughts (*i.e*., obsessions) that accompany compulsive behaviors are very much like the ruminations an addicted person experiences when having an urge or craving to do the behavior. However, Yates’ research [20] shows that these obsessive qualities of addictions are distinct from those in obsessive-compulsive disorders. Where obsessions are focused on unrealistic outcomes (e.g., a house fire will ensue if lights are left on), an addicted person ruminates about realistic negative outcomes of his or her behavior.

Compulsive disorders, as a type of anxiety disorder, are maintained primarily by negative reinforcement through anxiety reduction. Research shows that exercise addiction, like a compulsion, is maintained by its mood-altering effects. However, these effects extend beyond anxiety reduction to include lessening other negative affects including anger [21], depression, and boredom [11]. Unlike compulsive behavior, addictions are also maintained by enhancement of positive affect. In the case of exercise, there are the mood-improving effects of aerobic exercise [22] and increased self-esteem as the result of maintaining a disciplined regimen or improving appearance.

According to Goodman [23], addictions are distinguished from impulsive and compulsive behavior by their dual capacity to reduce negative affective states while also creating positive affects be it a rush or improved mood. In those cases in which exercise occurs only for its capacity to reduce anxiety, the problem may be better thought of as a compulsion. Christenson and colleagues [24] have made a similar argument for excess urge-driven buying that serves only to reduce anxiety.

### 2.3. Addictions that Co-Occur with Exercise Addiction

While the research on disorders co-occurring with exercise addiction is scant, estimates suggest that 15–20% of exercise addicted individuals are addicted to nicotine, alcohol, or illicit drugs [6]. For example, athletes who use stimulants such as amphetamines, cocaine or caffeine to improve athletic performance can become substance addicted [25,26]. However, when it comes to alcohol and nicotine use, some research has not supported a positive relationship [7]. Sussman, Lisha, and Griffiths [12] suggest that up to 25% of people with one addiction have another addiction. Buying addiction has been identified as common among the exercise addicted [13] while exercise addiction is common among individuals addicted to sex [27]. An analysis of the Shorter Promis Questionnaire (SPQ) that examines 16 potentially addictive behaviors finds that exercise tends to cluster with food disorders, caffeine use, and shopping [28]. A more recent study using the SPQ replicated these findings and added work addiction as another co-occurring disorder [29].

Eating disorders are the most common disorder to co-occur with exercise addiction. Approximately 39–48% of people suffering from eating disorders also suffer from exercise addiction [3,30,31].

The relationship between exercise addiction and eating disorders has significance for diagnosis and treatment. De Coverley Veale [9] distinguished primary and secondary exercise addiction; primary exercise addiction occurs in the absence of an eating disorder. Any weight loss is secondary to calories burned or if there is dieting, it occurs solely for the purpose of improving performance. However, for some people, the primary motivation for exercise is weight loss that occurs in the extreme. This kind of primary exercise addiction has been given a special name: *anorexia athletica* [31,32]. With secondary exercise addiction, exercise is paired with a co-occurring eating disorder. Exercise along with vomiting, laxatives, *etc*. serve to avoid the consequences of calorie consumption. Clinicians may think the latter is limited to women but this problem has been reported in college men [33].

Bamber, Cockerill, Rodgers, and Carroll [30] have argued that exercise addiction is always secondary to an eating disorder. In a questionnaire distributed to 194 women, they were unable to differentiate exercise addiction from eating disorders; in the absence of an eating disorder, participants’ exercise did not create the level of distress normally associated with an addiction. However, they focused solely on excessive exercise, which fails to take into account the criteria unique to an addiction, such as withdrawal and tolerance.

When exercise addiction and eating disorders co-occur, the danger is that only one problem will be treated. Often the eating disorder, as the better-known disorder, is the focus of treatment and the secondary exercise addiction remains hidden. Despite the improved relationship to food, the patient still does not gain weight, which is managed through an increase in the exercise regimen [2].

### 2.4. When is Frequent Exercise Not an Addiction?

One of the thornier issues in defining exercise addiction concerns how to distinguish healthy exercise from exercise addiction. In order to reap the health benefits of exercise, the behavior needs to be engaged in relatively frequently and for extended duration. In fact healthy exercise can share attributes of an addiction. There can be tolerance in which a person runs farther or lifts more weight before feeling gratified that the workout was worthwhile. Normal exercise does not preclude creating negative consequences in the form of physical injury or time taken away from other important activities.

Freimuth’s [2] clinical heuristic for distinguishing phases of addiction can be used to explore the distinctions between recreational exercise and exercise addiction. These phases help a clinician decide when a normal behavior is becoming addictive and when an addictive behavior is returning to normal. Each phase is broken into three components: motivation (referring to the person’s motivation for exercising in that stage), consequences, and frequency/control. The following example of Sally, based on a composite of several cases of exercise addiction, will be used to distinguish the phases:

Sally makes a New Year’s Resolution to “get in shape.” She begins going to the gym every morning before work. She enjoys how exercise has improved her strength and appearance but enjoys running the most because it helps her forget her worries and leaves her feeling relaxed. She begins running longer distances on the treadmill. As her endurance increases, she decides to train for a five-mile race with a group of other runners. She follows this program to the letter. After successfully completing the race, she feels wonderful and decides to continue the training regimen on her own, gradually increasing her distance. One day, while running on the treadmill, Sally twists her ankle. She has a severe sprain and her doctor recommends she stop running for the next few weeks. On the first day of refraining from exercise, Sally feels a little irritable. Over the next few days, she just doesn’t seem herself; she misses running and wonders if she is depressed. She begins to think the doctor over-reacted and decides to go to the gym just to lift some weights. She does this for two days but on the third day she cannot resist the urge to get on the treadmill; she runs until her ankle gives out.

#### 2.4.1. Phase One: Recreational Exercise

Recreational exercise primarily occurs because it is a pleasurable and rewarding activity [2]. This pleasure is represented by Sally’s enjoyment of the changes in strength and appearance from exercising. Another example would be a person who enjoys regular hikes because the experience of being in nature is pleasurable. Recreational exercise adds to the quality of life whereas addiction takes away from it [34].

Research shows that other sources of motivation in this phase are achieving health and fitness [35]. The behavior is under control; the person sticks to his or her schedule and is able to stop when planned. With recreational exercise, negative consequences are rare, unexpected, and usually a direct outcome of the exercise itself (e.g., a sore muscle, a sprained ankle).

#### 2.4.2. Phase Two: At-Risk Exercise

The recreational level provides the opportunity to discover whether a behavior is intrinsically rewarding, and herein lays the risk. Recreational exercise exposes a person to the potentially mood-altering effects of this behavior. Sally discovers that running has a special effect; it helps her escape her worries.

There is a great deal of evidence to show that exercise has mood-altering effects. Exercise serves to increase positive affect such as increasing self-esteem and decreasing the negative affect associated with depression and anxiety [22].

In some cases, these mood changes have been attributed to altered chemical functioning of the brain. Griffiths [36], after reporting a detailed case study of exercise addiction, proposed three possible biological mechanisms to connect improved mood and exercise:

- The Thermogenic Hypothesis: exercise increases body temperature, thereby reducing somatic anxiety. This decrease in anxiety is related to an increased temperature in certain brain regions [37];
- The Catecholamine Hypothesis: exercise releases catecholamines, which are strongly implicated in control of mood, attention, and movement as well as endocrine and cardiovascular responses linked to stress [38];
- The Endorphin Hypothesis: exercise releases endorphins, which are opiates that occur naturally in the body. This pleasurable experience of exercise may have unplanned consequences. With regular intense aerobic exercise, the increased endorphin production results in the brain down-regulating endorphin production. If this happens, the person will need to continue the exercise in order to maintain the natural balance in the brain [39].

Research with rats suggests another reason why exercise behavior, once it occurs at high frequency, may need to be maintained at high levels. Rats exposed to extreme exercise showed changes in the neurotransmitter dopamine that has been shown to play a significant role in other addictions. This kind of intense exercise in rats reduced the rewarding effect of other substances that also induce dopaminergic responses. Assuming this effect translates to humans, Adams [39] argued that it is possible that with reduced hedonic pleasure from other activities, a person may have to maintain the intense exercise in order to optimally activate reward circuitry of the mesolimbic dopamine system.

The mood-altering effects of exercise are available to all people, but not all people who exercise with increasing frequency and intensity ultimately become addicted to exercise. Those who do not develop problems with their exercise can be considered “highly engaged” in this behavior. Research on highly engaging behavior shows that it shares three common features with addiction: frequent thoughts about the behavior, positive feelings in response to the behavior, and tolerance (*i.e*., doing more of the behavior to get a good feeling) [2,40]. At one point, Sally is highly engaged in her running as she trains daily with a group, begins to increase her distance, and “feels wonderful” after completing her race.

There are a number of risk factors that help predict if a highly engaging behavior becomes fully addictive. These factors are biological (*i.e*., genetics and neurological) and psychological (*i.e*., negative peers, parental drug use, low self-esteem, juvenile delinquency, and low levels of social conformity) [41]. While these factors are known to influence addiction in general, there is evidence demonstrating that they play a role in exercise addiction.

Gapin, Etnier, and Tucker [42] linked frontal lobe asymmetry to increased risk of exercise addiction. Knowing that exercise serves to reduce negative affect, they demonstrated that women’s scores on the Exercise Addiction Inventory correlated with increased frontal lobe asymmetry that is a measure of negative affectivity.

On the psychological level, what distinguishes recreational exercise from exercise at-risk of increasing in frequency is the motivation. As LaRose, Lin, and Eastin [43] have shown with Internet use, an addiction is more likely when the primary motivation is *not* enjoyment from the activity but rather relief from stress or other types of dysphoria or to improve self-esteem. Thornton and Scott [35] have shown this effect for exercise; the likelihood of an addiction increases for those who exercise with the goal of escaping unpleasant feelings or transforming their appearance to improve self-esteem as compared to those who exercise with the goal of improving performance and fitness. The capacity of a behavior, like exercise, to serve a larger function, such as coping with unpleasant experiences, makes it more likely that the behavior will continue to increase in frequency and become problematic. Addiction is most likely to occur when the behavior is the primary or sole means of coping with internal distress [2].

In terms of observable signs, this important transitional phase from at-risk to early addiction is marked by periodic loss of control of the behavior that occurs for longer periods of time or is more intense than intended. Negative consequences increase in frequency. At the at-risk phase, these negative consequences are primarily a direct result of exercise (as distinct from later adverse effects which are more interpersonal in nature). Sally’s sprained ankle is a direct negative effect of her running.

#### 2.4.3. Phase Three: Problematic Exercise

Where recreational exercisers integrate their daily physical activity into their lives, those whose exercise is becoming problematic begin to organize their day around their excise regimen, which is becoming more and more rigid [44]. Another distinguishing feature of exercise at the problematic level is the nature of negative consequences. Where previously adverse effects arose directly from the behavior, at the problematic level secondary negative consequences are predominant. Secondary negative consequences include one’s own and/or another’s response to the adverse effects of exercise [2]. An example of a secondary negative consequence for Sally would be if her boyfriend began complaining about feeling she preferred running to being with him or if Sally became angry with herself for reinjuring her ankle.

Once in the problematic phase, the behavior continues despite having met the stated goal—much like the problematic drinker who continues to drink even after the desired stress relief from alcohol has occurred. In Sally’s case, after achieving the gratification of meeting her goal of running a five-mile race, she continues her diligent training regimen. Also common to the problematic level is that a behavior, once done socially, now occurs alone. Sally stops training with a group to train on her own.

Maintaining control over the behavior becomes more difficult in the problematic phase because, when the behavior ceases, withdrawal symptoms set in. The idea that behavioral addictions are associated with the classic signs of dependence-tolerance and withdrawal has been debated. At least with exercise, there is good evidence that aerobic exercise and intense use of large muscles is associated with changes in endorphins. If the body down-regulates endorphin production in response to exercise, the absence of exercise can be associated with withdrawal. Withdrawal from exercise has been demonstrated [45] and is illustrated by Sally’s irritability and not feeling like herself after her ankle injury. At this level, the behavior no longer occurs just for its mood-altering effects but also to remove withdrawal symptoms.

At the problematic level there are also signs that the behavior is becoming indiscriminant. Kohut [46] addressed this concept with regard to alcohol although it applies equally to other addictions: “for the alcoholic, alcohol is the important thing. It does not matter if it is good or bad bourbon, good or bad wine” (p. 118). While one form of behavior is preferred, at this stage, the exercise addict will try other forms of exercise when their preferred form is not available [36]. Sally begins doing more weight training while trying to wait for her ankle to heal.

#### 2.4.4. Phase Four: Exercise Addiction

The frequency and intensity of exercise continues until this behavior becomes life’s main organizing principle. The addicted athlete feels the physical rush and sense of gratification but continues to run further distances, lift more weights, or attend more gym classes. Consistent with the paradoxical nature of addiction, a behavior that began as a way to make life more bearable by facilitating coping ultimately makes life unmanageable. As the life of the addicted person revolves around exercise, the pleasure of the behavior recedes as the primary motivation becomes avoiding withdrawal symptoms.

Direct and secondary negative consequences continue to mount leading to tertiary negative consequences in the form of impairments in daily functioning and inability to meet role obligations. In the case of Sally, the loss of relationships due to her running and continued self-blame (secondary negative consequence that follows re-injuring her ankle) could result in a tertiary negative consequence of becoming clinically depressed.

## 3. Implications and Conclusions

### 3.1. Assessment and Treatment Implications

The Exercise Dependence Scale (EDS-R) was developed based on modification of the criteria for substance dependence [1,4]. The 21 items are responded to on a 6 point Likert Scale. The Exercise Addiction Inventory or EAI [10] represents another measurement tool based on Griffiths’ modification of Brown’s model of addiction [47]. Both the EDS-R and EAI have good validity and reliability. The EAI, with just six questions, is designed for use in medical and health care settings where assessment time is limited. Both scales yield three possible categories: asymptomatic, symptomatic, and exercise addicted.

When it comes to treating this addiction, abstinence from exercise may not be the required goal. Because exercise in moderation is considered a healthy habit, a typical treatment goal will be to return to moderate exercise. In some cases, a new form of exercise may be recommended; the runner becomes a swimmer. In other cases the person may continue to do the same form of exercise in a more controlled or moderate manner [34]. Whether moderating the original exercise behavior or replacing one activity with another, clinicians can use the attributes of the four phases of addiction as a way to help patients distinguish problematic or addictive exercise from moderate or recreational exercise.

There is scant literature on actual treatment of exercise addiction. Like most behavioral addictions, usually some form of cognitive-behavioral therapy is recommended [39]. One of the first issues will be to motivate clients for treatment given that qualitative studies of exercise addiction suggest that clients are insufficiently attuned to the adverse effects created by their behavior [44]. Without this recognition, exercise behavior is unlikely to be considered a problem meriting treatment. Once motivated, attention can turn to identifying and correcting automatic thoughts such as those related to the need to control the body [36] and the idea that exercise is always good even if it is done in a driven/obsessive manner [44]. Behavioral strategies, such as contingency management, that reward abstinence from a type of exercise or maintaining lower levels of a once addictive behavior, have also been recommended [39].

Whether assessing or treating exercise addiction it is always important to be attuned to the common co-occurring disorders, especially if it is an eating disorder or food related problem. If only the exercise addiction is treated, as exercise is reduced, a person will resort to increased bulimia or anorexic behavior in order to maintain low weight levels. Similarly the eating disorder specialist must attend to exercise behaviors in order that increased caloric intake is not compensated for by more intense exercise.

Clinicians who specialize in substance-related disorders and behavioral addictions such as sex, work, and shopping will want to be attuned to exercise addiction [13,27–29]. There is some evidence that exercise relieves withdrawal symptoms associated with cocaine addiction [48] but use of exercise for this purpose may open the way for an exercise addiction. In the case of behavioral addictions, as the primary addictive behavior decreases in frequency, it is possible that once moderate exercise becomes problematic as this behavior replaces the mood-altering functions of the initial addiction [27]. The need to be attentive to signs of exercise addiction is not limited to those who treat addictions. Some psychotherapy patients will use exercise as a primary form of mood regulation. The challenges of psychotherapy can lead to an increase in this behavior. Being aware of the phases of exercise addiction will help clinicians assess if recreational or at-risk exercise is becoming problematic or fully addictive.

Being attuned to such changes is essential for clinicians who recommend exercise to their patients. Recent research shows that exercise, with its ability to improve mood, can be an important adjunct to many different kinds of treatment [49] including eating disorders [17]. When exercise is recommended for its mood-altering effects, clinicians will want to be sure that the recommended regimen is not exceeded and remain attuned to any of the signs that exercise is becoming problematic.

### 3.2. Conclusion

The inclusion of behavioral addictions in DSM-5 [1] invigorates the quest to identify which kinds of addictive-like behaviors, other than gambling, should be included under this newly formed diagnostic category. To determine if exercise meets the criteria for an addiction, this paper set out to clarify the primary attributes and distinctive features of exercise addiction. In accomplishing this goal, topics also were addressed that inform clinicians about how to better identify and treat exercise addiction.

While there are several main approaches to identifying exercise addiction [7–11], this paper relied on Hausenblas and Downs’ [3,4] approach that is derived from a modification of DSM-IV TR [5] substance dependence criteria. Preference for their approach was largely shaped by the fact that these criteria will most likely be used to define behavioral addictions in future DSM versions. A number of attributes of exercise addiction (e.g., tolerance, obsessive thoughts) overlap with the exercise of committed athletes whose regimen involves intense exercise for long durations. However, as Freimuth [2] has argued, quantity/intensity of a behavior has never been a good measure of addiction. Instead, one can look to the four phases of addiction to distinguish such “highly engaging” exercise from addictive exercise.

Having presented one set of attributes for identifying exercise addiction, this paper addressed the question of whether exercise addiction is a distinct disorder or simply a manifestation of another disorder. Research was reviewed that demonstrated that exercise addiction, while a problem that commonly co-occurs with eating disorders, exists independent of an eating disorder [31,32]. Exercise addiction cannot simply be reduced to a compulsion or impulse control disorder either. Rather, like other high frequency behaviors that create adverse effects and are maintained by both positive and negatively reinforcement, certain patterns of exercise are best described as an addiction [23].

Although exercise addiction is not included in DSM-5, it is imperative that many kinds of health care providers become familiar with its attributes. The physician may see repetitive injuries and not recognize this as a sign that the compulsion to exercise prevents an injury from full healing. For psychotherapists, the patient committed to exercise may develop an addiction during the course of therapy if exercise is the primary means to manage the emotional demands of change. Addiction specialists also must remain attuned to the signs and symptoms of exercise addiction given its co-occurrence with substance use disorders and other behavioral addictions such as sex, work, and buying/shopping [13,25–27,29].

To date, treatment approaches for exercise addiction draw primarily from cognitive-behavioral principles used to manage other behavioral addictions. Whatever treatment is used, early identification of this problem will make it easier to treat [2]. To this end, the four phases of addiction were reviewed in order to highlight the attributes of an emerging exercise addiction. This system is not only useful for early identification but can be used when defining treatment goals and helping the patient distinguish recreational from addictive exercise.
